# Scaling up health interventions in resource-poor countries: what role does research in stated-preference framework play?

**DOI:** 10.1186/1478-4505-4-4

**Published:** 2006-03-30

**Authors:** Subhash Pokhrel

**Affiliations:** 1 School of Health Sciences and Social Care, Brunel University Osterley Campus, Borough Road, Isleworth, Middlesex, TW7 5DU, UK

## Abstract

Despite improved supply of health care services in low-income countries in the recent past, their uptake continues to be lower than anticipated. This has made it difficult to scale-up those interventions which are not only cost-effective from supply perspectives but that might have substantial impacts on improving the health status of these countries. Understanding demand-side barriers is therefore critically important. With the help of a case study from Nepal, this commentary argues that more research on demand-side barriers needs to be carried out and that the stated-preference (SP) approach to such research might be helpful. Since SP techniques place service users' preferences at the centre of the analysis, and because preferences reflect individual or social welfare, SP techniques are likely to be helpful in devising policies to increase social welfare (e.g. improved service coverage). Moreover, the SP data are collected in a controlled environment which allows straightforward identification of effects (e.g. that of process attributes of care) and large quantities of relevant data can be collected at moderate cost. In addition to providing insights into current preferences, SP data also provide insights into how preferences are likely to respond to a proposed change in resource allocation (e.g. changing service delivery strategy). Finally, the SP-based techniques have been used widely in resource-rich countries and their experience can be valuable in conducting scaling-up research in low-income countries.

## Background

Five major conditions – pneumonia, diarrhoea, malaria, measles and malnutrition- are responsible for 7 million child deaths each year globally [1]. The irony is that these deaths could have been avoided through the uptake of health interventions that are not only available but also proven to be cost-effective. It has been shown that about 63% of child deaths could be prevented if the coverage of essential health services, which include all cost-effective interventions, was increased to 95% [2]. Improving access to essential health services through "scaling-up" therefore seems to be a quick fix for bringing down mortality rates in the poorest counties. However, despite their potential to reduce mortality levels substantially [3,4], the coverage of essential health services continue to be low and millions of mothers and children continue to die [1].

Recently, an influential report by the Commission on Macroeconomics and Health has reinforced the need to extend coverage of essential health services in low-income countries while emphasizing that structural change in health services is needed to overcome the substantial barriers that exist in these countries [5]. Historically, the world has responded to a need to improve coverage of health services with a supply-driven approach but the success of supply-driven health planning and policy has been limited in improving health care demand [exhibit 1]. The case shown in the exhibit clearly demonstrates that there are two facets of coverage – (a) extending health services, a supply-side intervention ensuring that the services reach the population; and (b) promoting their uptake, a demand-side intervention ensuring that the needy use available services [6,7]. Although the line between these two facets can be blur, they are not the same issues and ignoring the "uptake" side in any scaling-up effort will result in inefficiency, as reflected by lower use of services and higher unit costs involved to deliver them. The question as to "how to scale-up interventions" will therefore remain a priority research and policy agenda in low- and middle-income countries in the next several years to come [8].

In response to this, there has been growing interest in understanding health seeking behaviour and patterns of utilization with a view to either changing them or catering better to them. The research in this area has been inter-disciplinary and rich [9]. One of the approaches to this type of research, often common in the health economics literature, is around disentangling the main drivers underlying individual's behaviour – looking at the pattern of their actual consumption of health services [9]. The disadvantage of relying on the actual consumption pattern is that we will not be able to observe how individuals valued the attributes of a health service and how this value affected their choice of care [10,11]. In addition, studies based on actual consumption patterns are not able to explain why a certain attribute of an individual (e.g. being a boy as against a girl) is likely to enhance their opportunities for early year investments such as that in health care [12]. Individual and social values, as reflected in the above two examples, are as important as determinants of uptake of services as other attributes such as health services' (for instance price and quality) and those of individuals' (such as levels of income and education). Unfortunately, although we have fairly adequate evidence on the effects of the latter on service uptake [6], we lack a clear understanding of the former [Exhibit 2]. The main aim of this paper is therefore to consider the potential benefits of using an alternative research approach to inform scaling-up strategies in low-income countries.

### Individual preferences and scaling up

Individuals do want health and health care but that is not all they want. While the individuals' wants are unlimited, their ability to pay for what they want is limited. Because individuals like and dislike things, they have preferences for one good or service to others. Preferences can be used as a measure of benefit (welfare) to the individual or society. By choosing a particular commodity or a service, individuals strike a balance between their wants and resources and thus their preferences reflect their own welfare. We can assume three things about individual preferences: (a) that we have preferences over every good and service, (b) that any outcome of using a good or a service is at least as good as itself, and (c) the preferences are transitive, i.e. if a service A is preferred to B and B to C, A is preferred to C [13].

These concepts around preferences thus provide a platform to compare two or more possible outcomes, e.g. using a doctor's service versus a traditional healer's rituals in the event of an acute illness. By estimating a numerical representation of preferences (known as 'utilities'), it is possible to isolate the effects of several factors associated with individual behaviour. These factors could be characteristics specific to (a) individuals, e.g. age, sex, income, current health status; (b) service providers, e.g. quality and price of care; or (c) the values, e.g. cultural norms such as the value placed on boys in some societies or belief and attitudes on service delivery processes [12,14-16]. Once we disentangle these factors, appropriate policies can then be formulated. However, it is important to note that these would-be policies might have different underlying assumptions depending upon the context in which they are to be implemented. In high-income countries, for example, research is being carried out to inform policy on how best we can incorporate public preferences in the delivery of health services [11,15]. In resource-poor countries, on the other hand, decision makers need policies that would alter effectively the individual preferences which are deemed to be detrimental, e.g. not seeking care in the event of an illness or ignoring a child's need for care if the child is a girl [12,17]. Effectively altered preferences, in principle, should bring about positive changes in health service uptake. Regardless of the context, the theoretical basis for a preference-based research remains the same and understanding individual preferences does contribute to a predefined policy objectives such as improved health outcomes either via refinement in health service delivery as in high-income countries or via scaling-up of interventions as in resource-poor countries [Exhibit 3].

### Revealed versus stated-preference

The preference of an individual (or a provider or a society at large) can be studied in two paradigms- revealed preference (RP) and stated-preference (SP). The revealed preference paradigm draws on Samuelson's seminal article [18] and involves the exploration of people's preferences as revealed through their actions in markets, specifically related to the value of interest. Examples of such methods include travel cost method [14,19] and hedonic pricing technique [19,20]. Although these methods differ in terms of how the price variable enters individual's utility function (a numerical representation of their preferences), the essence of both methods lies in the concepts of opportunity costs and trade-off. That is, people weigh their costs and benefits and make consumption decisions accordingly. Their actual consumption pattern therefore reflects their preferences.

The alternative pathway involves asking the same individuals to state their preferences in hypothetical (or virtual) markets. Individuals are given different hypothetical scenarios (e.g. two different ways of delivering antenatal care) and they are asked to state their valuations of these options or choose the option that they prefer. The methods that follow this strategy are collectively known as "stated-preferences" (SP) techniques [10,21]. The two-best known SP techniques are the contingent valuation method (CVM) and discrete choice experiments (DCE). The CVM asks for the valuation of a particular intervention either directly or relative to other intervention [21-23]. The values derived from this method are known as the "willingness-to-pay" or WTP for a specific intervention and reflect the benefit of the intervention in question. The DCE (alternatively known as conjoint analysis), on the other hand, is a rigorous method of eliciting individuals' preferences in that 'it allows estimation of the relative importance of different aspects of care, the trade-offs between these aspects, and the total satisfaction or utility that respondents derive from healthcare services'[11]. As the name suggests, in this technique a number of hypothetical scenarios are formulated and the individuals are asked to give a discrete choice (e.g. I prefer care A to care B). The DCE also allows an indirect estimation of WTP values relative to different aspects of care, e.g. how much individuals are willing to pay for reduction in waiting time [16]. Detailed description of these methods is provided elsewhere [10].

It is important to note that there are other techniques too which are SP-based and have been in use in economic evaluation for a long time, e.g. bidding game, standard gamble, time-trade off and person trade-off. A good overview of these methods is provided in [21]. More recently, qualitative analysis has shown potential to be one of the SP-based techniques in its own right [24-26]. However, it has certain limitations which confine it to play a complementary role to other preference (both revealed- and stated-) techniques [21,27].

### Benefits of stated-preference

There are a number of reasons why stated-preference is more useful than revealed-preference in understanding health care choices. The SP framework, by virtue of its design, is able to point out explicitly the mechanism by which people trade off different aspects of care when they make a health care decision [10]. In the RP framework, because we are left with the data on the actual consumption and health outcomes only, we are not able to see this trade-off happening. SP techniques, on the other hand, extend beyond health outcomes and focus on the process of care providing a more holistic approach to study health care choices [24]. Putting this into the context described in Exhibit 1, we may say that people in Nepal might have valued other unobserved aspects of care while making child care choices. Until we understand what these aspects are and their relative values from individual's perspectives, the current scaling-up efforts will not result in the desired uptake of services. While we can be numerically better off in terms of service outlets, their sub-optimal use does not allow us to achieve the targeted coverage. Without achieving targeted coverage, it is impossible to reduce the mortality level to the extent promised by cost-effective interventions under their optimal level of uptake [2,3]. From a more technical viewpoint, SP techniques place service users' preferences at the centre of the analysis and because preferences reflect individual or social welfare, these techniques are more likely to flag up policy leads on increasing social welfare (improved coverage in this example) [24].

From scaling up perspectives, another advantage of SP over RP framework is that the SP data are collected in a controlled environment which allows straightforward identification of effects (e.g. that of process attributes of care) and large quantities of relevant data can be collected at moderate cost [10,11,15,16,21]. In addition to providing insights into current preferences, SP data also provide insights into how preferences are likely to respond to a proposed change in resource allocation. Last but not least, it may not be possible to infer consumer preferences or value from RP data because many aspects of health care are not traded explicitly in markets, have public good characteristics (e.g. vaccination services) and consumption is free or heavily subsidized at the point of service via government provision of care and universal or private insurance if they exist [28].

Although health economists do not seem to agree that qualitative methods can be an SP-based technique on its own right [21], the value of having qualitative methods to complement an RP technique must be recognized [27,29]. Qualitative methods facilitate a more in-depth inquiry of the topic at hand and allow the researcher and the respondents to 'fully explore the rich tapestry of causation and interaction that can explain personal and social behaviour' [30]. Putting this into the context described in Exhibit 2, qualitative methods could be more useful in disentangling why just 'being born as a boy' leads to more opportunities for early life interventions to men. Clearly, this has implications for non-discriminatory scaling up of health care interventions in countries where substantial degrees of gender-bias in health care use exist [12,31].

## Discussion

It is important to note that all of the advantages of SP techniques described above do not make them 'stand-alone' techniques to answer all questions relating to scaling-up issues. The RP framework has its own advantages too. For example, in the example given in Exhibit 2, it is the RP technique that has demonstrated the effect of gender role on child health care decisions in Nepal providing implications for scaling-up policies, although offering explanations for why the bias exists is beyond its scope. The recent trend seems to be to combine both SP and RP approaches in such a way that the two approaches inform one another to fulfil the specific aims of a particular study [29,32]. It is possible to combine actual usage data with stated-preference data [32] or revealed consumption pattern with qualitative responses [33]. The combination of different methods in a single study however raises a number of challenging issues, e.g. that of triangulation (i.e. how can we make sure that the data collected through various methods are coherent) because the triangulation methods have not been adequately established [29]. Despite methodological challenges, the combination approach might turn out to be more informative than using data from a single source [10,27,29]. These trends are positive and therefore should be welcome in scaling-up research.

There are a number of problems associated with individual preferences and basing scaling-up research on preference paradigms is likely to be problematic too. Relying on what consumers say they will do (stated-preference) compared with observing what they actually do (revealed-preference) reflects a healthy scepticism [22]. Moreover, we will need to measure preferences using SP-based techniques as if they would reflect revealed preferences. This can be hampered by measurement biases such as the extent of information given to respondents (information bias) or the starting value of a bid in CVM (setting bias) [10,21]. Also important is the fact that preferences are not stable over time and current preferences can change if opportunities for learning, information or policy change arise. This makes the validation of preferences (i.e. comparing stated-preference with revealed preference) both impossible and irrelevant (perhaps combining the two approaches as discussed earlier is a good alternative). However, this dynamism in preferences provides lots of opportunities for scaling-up because understanding current preferences and how they would respond to a proposed change in resource allocation (e.g. changing service delivery strategies) is the information that policy makers are looking for in order to improve coverage of cost-effective interventions in low-income countries.

This paper flags out the potential benefits of using SP techniques in research aimed at informing scaling-up strategies in low-income countries. However, it is important to note that these techniques have long been applied in public health research but to answer different questions. The evolution of time trade-off techniques, for example, dates back to the 1970s, and application of 'standard gamble' methods to 1980s [see [21] for an overview]. These techniques have been used to derive a value for an individual's health status (technically known as health-state preference values) and used to calculate the total benefits an intervention would offer. Recent applications of stated-preference techniques are concerned with complementing an intervention's cost-effectiveness values with information on how patients might value that care or providing an alternative to traditional cost-effectiveness approach [15,16,23,34]. The SP-based techniques, particularly the DCEs, have been proved to be an excellent tool to inform changes in the current delivery strategy that will improve the service's uptake [11,15,16]. So far, this type of research has predominantly been carried out in resource-rich countries where the policy question is: how do we take into account people's views in the delivery of health services? [11,15,16]. The same question is being asked in low- and middle-income countries but in a different context and with a different policy objective- how do we change people's current preferences that are leading to much lower use of very cost-effective health interventions? [6-8,12]. In other words, the question as to how we can devise our delivery strategy in such a way that it will result in an improved coverage of essential health services (scaling up) must be answered [8]. The gap between delivery and demand must be understood sufficiently [7]. Although policy questions and objectives differ in the two settings, the theoretical concept of preferences remains the same. Therefore, SP-techniques, given their wider and successful application in rich countries, can be valuable tools in scaling-up research in low- and middle-income countries. So will be the experience gained by rich countries on their application.

## Conclusion

This paper argued that scaling-up of health services has two facets- one is 'extending the availability of cost-effective interventions' to the population (coverage) and the other is 'increasing the level of demand' for these services (uptake). While improving supply of interventions is a necessary condition in any scaling-up process, understanding the uptake of services is critical. One of the approaches to understanding uptake is to analyze individual preferences. This can be done in two ways- looking at actual health care consumption pattern (revealed preference or RP) or asking the same economic agents to state their preferences in hypothetical markets (stated-preference or SP). The paper explores the benefits of using SP techniques in scaling-up research. Since SP techniques place service users' preferences at the centre of the analysis SP techniques are more likely to be helpful in devising policies improve service coverage. Further, the SP data are collected in controlled environments and thus allows straightforward identification of effects (e.g. that of process attributes of care) and large quantities of relevant data can be collected at moderate cost. The SP data not only provide insights into current preferences, they also provide insights into how preferences are likely to respond to a proposed change in resource allocation (e.g. changing service delivery strategy). The SP-based techniques have been used widely in resource-rich countries and their experience can be valuable in conducting scaling-up research in low-income countries.

## Exhibit 1: The gap between delivery and demand in Nepal

**Figure 1:**
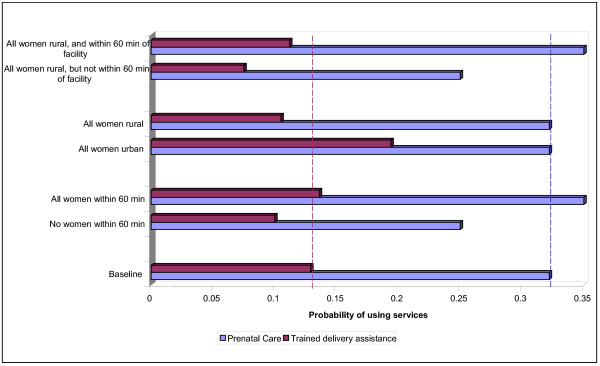
**Simulated impact of location on probability of using prenatal care and trained delivery assistance in Nepal. **Purple bar – Prenatal care. Red bar – Trained delivery assistance. This graph is based on the data provided in [36].

Following the national health policy 1991, His Majesty's government of Nepal (HMG/N) has invested substantially in the development of primary health care infrastructures in rural areas in a bid to improve service coverage. The number of sub-health posts, for example, increased more that 15-fold between 1990/91 and 1999/00- from 200 to 3179 [see Table 1]. With this supply-driven approach, access to health care has been improved significantly but the gap between delivery and demand is still wide. For example, a study found that introducing outreach clinics to all communities would be expected to increase the maternal and child health (MCH) service use index by 22 percent while if each community had better physical access to services from both outreach clinics and fixed facilities, the MCH service use index would increase by 32 percent [35]. However, it is critically important to note that individuals in this sample valued outreach clinics for other reasons, not necessarily because these clinics tend to reduce the physical distance they needed to travel in order to receive the care. As shown in Figure 1, another study using the same sample confirmed that physical access to services had only a modest impact on health care use [36]. Within this supply-driven health policy, it is therefore critical to understand how individuals value other attributes of care vis-à-vis close proximity to it. Hence, what we need to know before we embark upon developing strategies to scale up interventions is perhaps the answer to the following question: how and to what extent do individuals trade off between physical distance and other attributes of care?

**Table 1:** Extension of health infrastructure vis-à-vis change in population and health status in Nepal 1984–2004.

	**1984/85**	**1991/92**	**1995/96**	**1999/00**	**2003/04**
**Health infrastructure**					
***Service outlets***					
Hospitals	80	113	82	83	83
Primary Health Centre*			79	160	180
Health centres	26	18	17	13	10
Health posts	744	816	775	711	700
Sub-health posts	-	200	2597	3179	3141
***Hospital beds***	3522	4798	3604	5190	5250
***Human resources***					
Doctors	602	1497	872**	1259**	1259
Nurses	2109	2986	4606	4655	10099
Health assistants	795	3461	5152	5295	7491
Maternal and child health workers	3345	20442	3187	3190	3190
Others (trained birth attendants, female community health volunteers)	-	-	55109	62546	62546
**Health Status**					
Infant mortality	126	110	96	83	64
Under-5 mortality	187	153	131	117	91
Life expectancy at birth	49.1	52.0	54.6	57.3	59.8
**Population**	16.2	18.1	20.4	23.0	25.2

- Data not available. Sources of available data: Sources: [39-41].

* Established after 1991 Health Policy. **Includes government-employed doctors only.

## Exhibit 2: Limitation of revealed-preference in a study on gender role in Nepal

One of the important limitations of revealed-preference framework (i.e. looking at actual health care consumption) is its inability to capture the 'process' by which these preferences evolve [9]. A recent study used a four-step construct of household decision making in which the decisions to report an illness of the child, to seek an external help, to choose a specific provider from the available ones, and to spend a certain level of money to treat the child, were assumed to have been made in a hierarchy [7]. This was an innovative attempt to capture the process by which preferences evolve, as the construct was drawn on the qualitative way of looking at the health-seeking behaviour; the focus however was on the analysis of actual consumption pattern (revealed preference). The study indicated that households' preferences for health seeking, as observed in illness-perception rates, were significantly higher if the ill child was a boy (comapred to a girl). The degree of this bias got larger as households chose to seek care for the child and decided to spend money to treat him [12]. Although possible explanations of this behaviour were discussed, the study was not able to answer *why *this differential actually exists. The authors flag out potential uses of stated-preference techniques to fill in this gap [12].

## Exhibit 3: Where do we begin in scaling-up research?

A recent research [9] has found that the level of illness perception in Nepal was extremely low (10%), consistent with several other studies done elsewhere in low-income countries. The illness perception is important because people in low-income countries tend to demand health care services only when they perceive themselves as ill [37]. Low demand of services such as treatment of malaria and tuberculosis has spillover effects as well [38]. Policy makers need the answer as to how we can increase the illness perception rate here. This is important because the analysis of consumption data based on this level of illness perception rates leads to policy recommendation such as improving the supply of cost-effective health interventions and lowering the costs of care to households [17]. However, when the illness perception rate is as low as 10%, these supply-driven policies will affect only a very small portion of the population. Since we can not assume that in poor countries like Nepal basic health care need can be as low as 1 in 10, such policies alone cannot address scaling-up efforts. Individuals' preferences therefore matter and to improve scaling-up of interventions, we might have to begin with a research looking at how individuals in poor-countries value their current health status vis-à-vis how they value the current mode of health service delivery.
